# Noninfectious Cloudy Peritoneal Effluent in a Peritoneal Dialysis Patient with Mantle Cell Lymphoma

**DOI:** 10.7759/cureus.3413

**Published:** 2018-10-04

**Authors:** Darlene Vigil, Michael D Reyes, Sherryl Polak, Yijuan Sun, Lisa Blacklock, Antonios H Tzamaloukas

**Affiliations:** 1 Nephrology, Raymond G. Murphy VA Medical Center and University of New Mexico School of Medicine, Albuquerque, USA; 2 Pathology, Raymond G. Murphy VA Hospital, Albuquerque, USA; 3 Internal Medicine, Raymond G. Murphy VA Medical Center, Albuquerque, USA; 4 Internal Medicine, Raymond G. Murphy VA Medical Center, University of New Mexico School of Medicine, Albuquerque, USA; 5 Radiology, University of New Mexico School of Medicine, Albuquerque, USA; 6 Internal Medicine, University of New Mexico School of Medicine, Albuquerque, USA

**Keywords:** peritoneal dialysis, malignancy, mantle cell lymphoma, cloudy peritoneal dialysate

## Abstract

A 77-year-old man on peritoneal dialysis (PD) presented repeatedly with cloudy spent dialysate containing an elevated mononuclear cell count. He had mantle cell lymphoma diagnosed by colonic polyp biopsy two years before the start of PD. The first episode of cloudy dialysate was treated for peritonitis. However, the culture of the peritoneal fluid was negative and the mononuclear cells were proven to be atypical lymphocytes of the mantle cell lymphoma variety. In addition to the peritoneal effluent, atypical lymphocytes were also found consistently in the patient’s blood samples and once in his right pleural effusion. The patient exhibited high peritoneal transport status and clinical features of volume overload raising the question of alterations in the peritoneal transport processes in PD patients with malignancies involving the peritoneal membrane. Distinction between infectious and noninfectious cloudy dialysate and the potential of changes in the peritoneal membrane transport mechanisms are issues that should concern the care of PD patients with cloudy dialysate containing malignant cells.

## Introduction

Peritonitis is the usual cause of cloudy spent peritoneal dialysate in patients on peritoneal dialysis (PD) presenting with or without abdominal pain, fever, nausea, vomiting, or diarrhea. However, the source of cloudy dialysate can be other than infectious. In an informative review, Rocklin and Teitelbaum analyzed the pathogenesis and diagnosis of noninfectious causes of cloudy peritoneal dialysate [1]. The authors distinguished two general sources of cloudy dialysate, conditions causing an increased output of cells in the peritoneal cavity and those leading to intraperitoneal accumulation of compounds causing cloudiness of the dialysate (e.g., chylous ascites). 

Cells causing cloudy peritoneal dialysate of noninfectious nature can be leucocytes, red blood cells, or atypical cells [1]. Malignant cells or malignancies causing increased mononuclear cell counts in the dialysate as a cause of noninfectious cloudy dialysate have been reported in a small number of patients [2-8]. We report a case of cloudy peritoneal dialysate due to the presence of mantle cell lymphoma cells. This case report illustrates issues created by cloudy dialysate containing malignant cells. 

## Case presentation

A 77-year-old man on PD for five months transferred his care to our unit in January 2018. He had a history of type 2 diabetes mellitus for 40 years and had been followed for chronic kidney disease presumed to be secondary to diabetic nephropathy since 2009. In November 2015, histological examination of colonic polyps removed during routine colonoscopy revealed the presence of prominent nodular aggregates of atypical small to medium size lymphocytes positive for b-lymphocyte antigen CD20 (CD20), cyclin D1, and B-cell lymphoma 2 (BCL-2), with weak expression of lymphocyte antigen CD5 (CD5) and approximately 20% of the lymphoma cells staining for cellular proliferation marker Ki-67 (Ki-67). The histological diagnosis of mantle cell lymphoma was made. He had no symptoms consistent with lymphoma. Complete blood count revealed modest anemia, and normal white cell and platelet counts. The blood smear did not contain abnormal lymphocytes at that time. Serum lactic dehydrogenase (LDH) level was in the normal range at presentation and throughout the course of his disease. Positron emission tomography-computed tomography (PET-CT) showed scattered metabolically active enlarged lymph nodes in both axillae, mediastinum, and around the upper abdomen around the pancreas. Diffuse metabolic activity was also detected in the spleen, which was enlarged. The patient, who lives at a distance from Albuquerque, New Mexico, chose to be followed by an oncologist closer to his home who suggested postponing the start of antineoplastic treatment until the appearance of signs of disease progression.

Clinical manifestations of lymphoma and signs of disease progression on subsequent surveillance PET-CT studies were absent initially. However, renal function, which was worsening slowly up to that point, worsened rapidly in the second half of 2016, and he was placed on hemodialysis in a dialysis unit close to his home. A percutaneous kidney biopsy performed in December 2016 showed diffuse proliferative (class 4) lupus nephritis, which did not respond to a four-month course of prednisolone and mycophenolate. He had no past medical history of lupus.

In August 2017, he changed his dialysis modality to PD. For the first five months of PD, he was followed in the dialysis unit close to his home. The status of peritoneal transport was investigated by a standard peritoneal equilibration test performed approximately two months after the onset of PD. This test showed a four-hour dialysate-to-plasma ratio for creatinine (four-hour D/P creatinine) of 0.81 which is consistent with high-average transport. Adequacy of azotemic substance removal was tested by standard weekly total (peritoneal plus renal) fractional urea clearance (Kt/V urea) which was 1.81. Kt/V urea values ≥ 1.70 weekly are considered adequate.

On his first visit to our PD unit he had no abdominal symptoms but the spent peritoneal dialysate was cloudy. Dialysate cell count was as follows: red cells 135/mm^3^, nucleated cells 693/mm^3^ (neutrophils 5%, lymphocytes 79%, macrophages 15%, mesothelial cells 1%). The lymphocytes had an atypical morphological appearance consistent with the diagnosis of mantle cell lymphoma. At the same time, blood hemoglobin was 13.1 g/dL, hematocrit 38.0%, platelet count 134 × 103/mm^3^, and white cells 11.8 × 103/mm^3^ with 26% neutrophils and 69% lymphocytes disclosing atypical features. He was started empirically on intraperitoneal vancomycin with the presumptive diagnosis of peritonitis. Spent dialysate culture, however, was negative. On each subsequent PD clinic visit, spent peritoneal dialysate contained atypical lymphocytes. Atypical lymphocytes were also found repeatedly in his blood samples.

Recent PET-CT scans showed modest enlargement of several metabolically active lymph nodes and spleen. Blood hemoglobin and hematocrit had decreased to 9.9 g/dL and 30.4%, respectively, in mid-July 2018. At the same time, white blood cell count was 18.6 × 103/mm^3^ with 81% atypical lymphocytes. Fluid retention, manifested by peripheral edema and moderate right pleural effusion, was present in all his visits to the PD clinic, but became symptomatic with respiratory distress in early July 2018 when he was admitted with the presumptive diagnosis of pneumonia. This diagnosis was not confirmed. However, paracentesis of the right pleural effusion revealed cloudy fluid with 12142/mm^3^ red cells and 3505/mm^3 ^nucleated cells, 9% of which were neutrophils and 74% lymphocytes with atypical appearance. In the same sample, protein concentration was 2.6 g/dL, LDH 723 U/L, and glucose concentration 107 mg/dL while serum glucose was 151 mg/dL.

The following serum laboratory values were obtained recently: albumin 2.4 g/dL, LDH 432 U/L (normal range 300-670 U/L), and antinuclear antibodies negative. Serum light chain analysis showed kappa chains 330.5 mg/L (normal range 3.3-19.4 mg/L), lambda chains 175.3 mg/L (normal range 5.7-26.3 mg/L), and kappa/lambda ratio 1.89 (normal range 0.26-1.65). Weekly total Kt/V urea was 1.63. The decline over the previous value of Kt/V urea of 1.81 was due to loss of residual renal function. A repeated peritoneal equilibration test showed a high transport state with a four-hour D/P creatinine of 0.86 and ultrafiltration volume of 265 mL. The value of the Kt/V urea was not in the adequacy of dialysis range and the high transport status diagnosed by the last peritoneal equilibration test is known to be associated with fluid retention. The prescription of PD was changed with addition of one daily exchange containing icodextrin to the previous five nocturnal exchanges containing dextrose. In the last PD clinic visit of the patient in August 2018, his edema was substantially reduced and he had not had symptoms related to lymphoma. He is currently discussing the potential advantages and disadvantages of chemotherapy with his oncologist.

**Figure 1:**
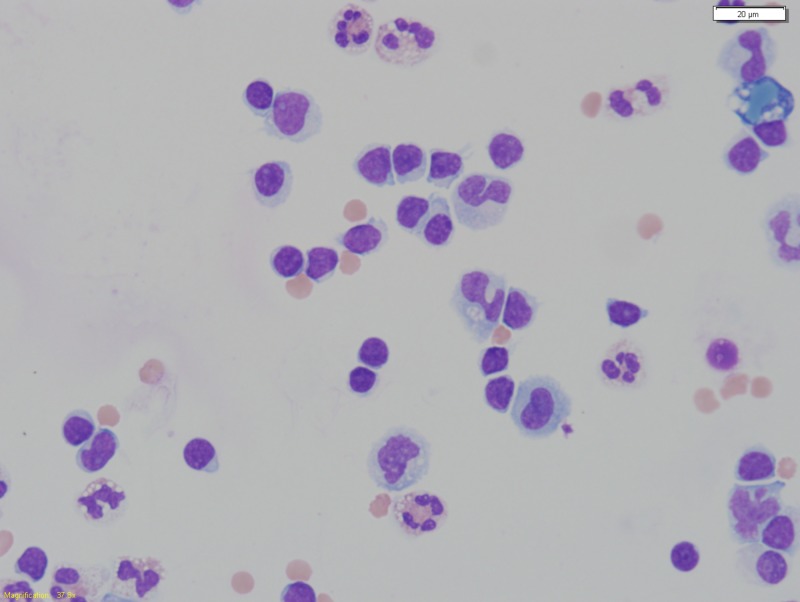
Peritoneal fluid. The figure shows atypical small to intermediate size lymphocytes with mature chromatin, subtle nuclear irregularities, scant to moderate basophilic cytoplasm and variably prominent nucleoli. These morphologic findings are compatible with mantle cell lymphoma.

**Figure 2:**
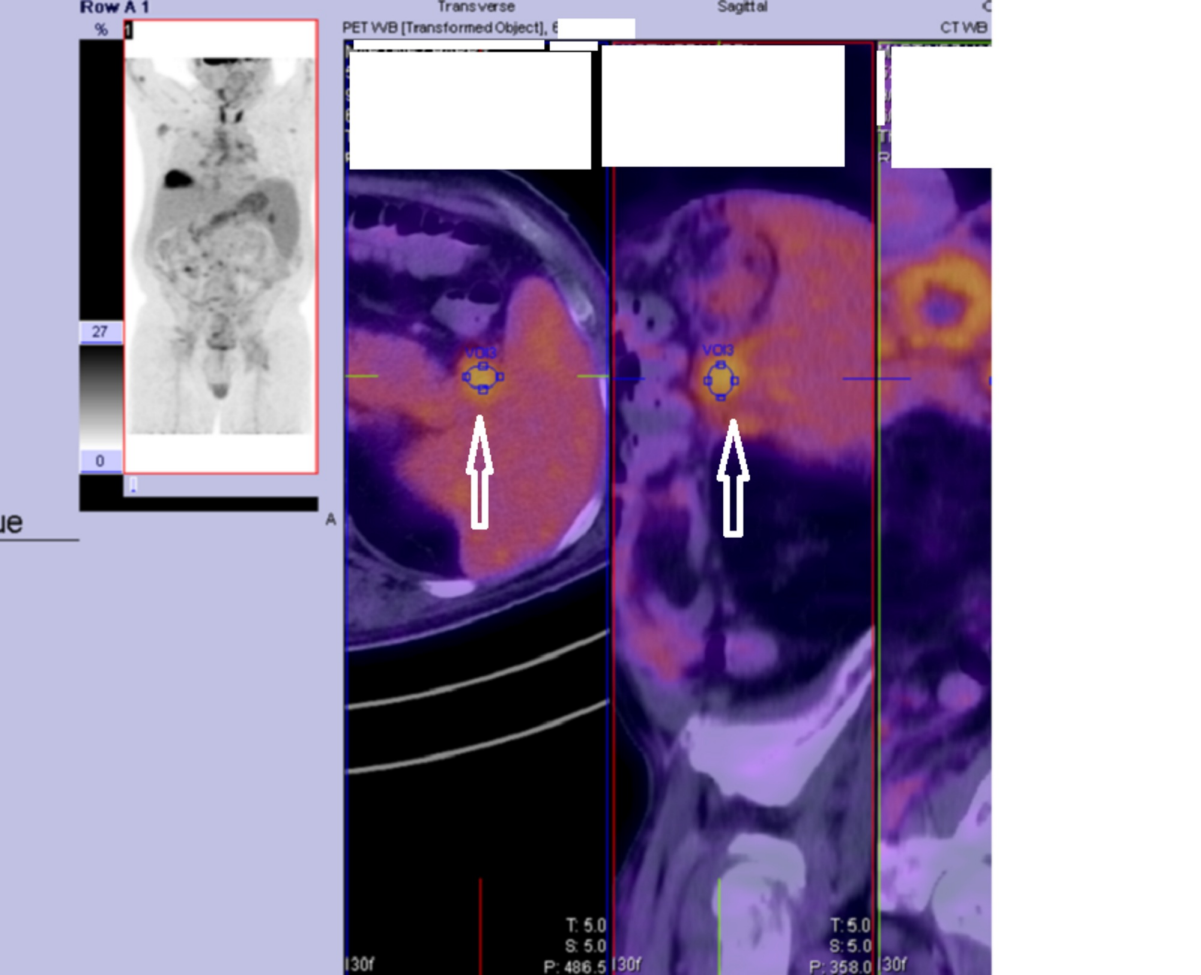
Abdominal PET-CT. PET-CT, positron emission tomography-computer tomography. Arrows:  enlarged lymph nodes with increased metabolic activity adjacent to an enlarged spleen, which also exhibits increased metabolic activity.

Figure 1 shows atypical lymphocytes and a few neutrophils in his peritoneal effluent. Figure 2 shows a recent PET-CT image with enlarged and metabolically active lymph nodes and an enlarged and metabolically active spleen.

## Discussion

The management of PD in patients with malignant cells or mononuclear cells associated with malignancy in the peritoneal dialysate faces two questions: (a) is peritonitis present? and (b) has the malignancy altered the peritoneal solute and water transport mechanisms? In conjunction with the other reported cases [2-8], our case report sheds some light on these questions. Table 1 summarizes the key findings of these cases. Including our patient, age of the patients ranged between 47 and 77 years, while four patients were women, three were men, and one report did not provide the gender of the patient [7].

**Table 1:** Reports of cloudy dialysate containing malignant cells or mononuclear cells associated with malignancy. PD, peritoneal dialysis; ESKD, end stage kidney disease.

Reference	Histology	Clinical manifestations	Outcome
[2]	B-cell lymphoma	Relapsing disease x 10 years. Constipation, abdominal discomfort, mild fever, culture-negative cloudy dialysate. Serum creatinine and ultrafiltration unchanged	Continued PD. Listeria peritonitis in two months leading to death from sepsis
[3]	Renal carcinoma	15 episodes of culture-negative cloudy dialysate containing neutrophils and treated with antibiotics. Nephrectomy	Continued PD. Two peritonitis episodes, but no episode of sterile cloudy dialysate post-nephrectomy
[4]	Endometrial carcinoma	Hysterectomy two years in the past. Abdominal discomfort, leg swelling, pain	Continued PD. Sudden death in two weeks
[5]	B-cell lymphoma large cells	Culture-negative, painless, cloudy dialysate. Malaise, anorexia. Malignant cells in dialysate and needle biopsy of lymph node	Palliative treatment. Death in two weeks
[6]	Prostatic carcinoma	Painless cloudy dialysate with atypical cells growing coagulase negative Staphylococcus and treated with antibiotics. Three more similar episodes in five months. Prostatectomy	Continued PD. Death nine months post prostatectomy probably from coronary syndrome
[7]	B-cell lymphoma large cells	Diagnosed by nasal mass biopsy. Two weeks later, presentation with decreased ultrafiltration, abdominal pain, and culture-negative dialysate containing 80% mononuclear cells, and treated with antibiotics	Chemotherapy. Continued PD
[8]	Hodgkin’s lymphoma	Several relapses. ESKD secondary to chemotherapy. Asymptomatic cloudy dialysate leading to diagnosis of new relapse	Switched to hemodialysis. Bone marrow transplantation
This case	Mantle cell lymphoma	Diagnosed by colonic polyp biopsy 2 years prior to starting PD. Culture-negative cloudy dialysate containing atypical cells on various occasions. Received antibiotics once. Malignant cells also in blood, pleural fluid. High peritoneal transport, low Kt/V urea due to loss of residual renal function	Continues PD for one year. Considering chemotherapy in view of apparent disease progression

The question of presence or absence of peritonitis in the presence of cloudy dialysate was addressed in several case reports summarized in Table 1. This question should be raised regardless of whether there have been prior episodes of culture-negative cloudy dialysate containing malignant cells or the patient does not have clinical manifestations suggesting peritonitis. Informed and timely collaboration between clinicians and laboratory has a chance of reducing errors in this case. Specifically, rapid identification of the type of cells in the spent dialysate could lead to timely initiation of antibiotics if the number of neutrophils has increased substantially over previous determinations or to the decision to withhold antibiotics if the neutrophil count in the dialysate has remained low. A positive Gram stain of the dialysate can help the decision to start antibiotics while a negative Gram stain has no diagnostic value. Culture of the dialysate is imperative. However, even in the best of circumstances diagnostic errors can occur. The consequences of not treating a peritonitis episode or treating it late are potentially worse than using antibiotics unnecessarily. Consequently, monitoring of the clinical status and the peritoneal fluid of patients who have not received antibiotics is critical. The option of changing to hemodialysis should also be discussed with the patient.

The second question raised by the presence of atypical cells in the dialysate is whether the malignant process has affected peritoneal ultrafiltration and/or uremic solute removal. This question, which is relevant for patients with longer life expectancy, e.g., those with lymphoma, has not received adequate attention as can be seen in Table 1. Although several patients continued PD for some time after the diagnosis of malignant processes leading to the presence of atypical cells in the peritoneal dialysate, repeated formal testing of the peritoneal transport mechanisms was reported only in our patient. The possibility that either the malignant process or its treatment alters the peritoneal transport processes should be kept in mind. Periodic measurement of Kt/V urea is routinely performed in PD patients. Monitoring of the peritoneal ultrafiltration by frequent clinical evaluation for fluid overload is done routinely. However, periodic performance of peritoneal equilibration test, which is routinely not done repeatedly, should be considered in patients with malignant cells in the peritoneal dialysate.

Lymphoma was responsible for five of the eight patients described in Table 1. In addition to cloudy dialysate containing atypical cells, lymphoma also has been the cause of other clinical manifestations in PD patients [9-11]. One PD patient with acquired immunodeficiency syndrome (AIDS) developed purulent discharge from a rapidly enlarging mass at the site of the PD catheter insertion. Biopsy of the mass revealed plasmablastic lymphoma [9]. A second patient on PD developed two masses in the anterior abdominal wall. These masses, the size of which increased rapidly, were initially thought to represent a PD catheter leak in the abdominal wall, but were proven to be tumors containing large cell B-cell lymphoma [10]. Finally, a patient placed on PD after failure of a renal transplant performed many years earlier was admitted with a clinical picture of sepsis, but was found by bone marrow biopsy to have a late-onset T-cell lymphoma representing post-transplant lymphoproliferative disorder (PTLD) and died within a short time [11].

Our patient had mantle cell lymphoma, which is a B-cell lymphoma arising from cells in the mantle zone of lymphoid follicles expressing the CD5 antigen [12]. Jares and co-investigators provided a detailed analysis of the pathogenetic pathways associated with the varying clinical manifestations of mantle cell lymphoma [13]. The primary mechanism leading to the development of this type of lymphoma consists of a t(11;14)(q13;q32) translocation causing an overexpression of cyclin D1 [13]. The clinical manifestations may vary from indolent, as they were in our patient at the diagnosis of lymphoma, to severe [14-15]. The low percent of lymphoma cells positive for the Ki-67 proliferation index found in our patient is an indicator of better prognosis [16]. Features of mantle cell lymphoma that are relevant to the manifestations of the disease in our patient include a high frequency of gastrointestinal tract involvement [17] and the indolent finding of the disease in colonic tissue followed by disseminated disease several years later [18]. Circulating atypical cells are frequently encountered and in the most aggressive cases can behave as acute leukemia [19].

Treatment is in general less effective in patients with mantle cell lymphomas than those with other types of lymphomas [14-15]. The current therapeutic approaches are combination chemo-immunotherapy with cytarabin and rituximab followed by autologous stem cell transplantation in young patients and combination immunotherapy followed by the kinase inhibitor ibrutinib in older patients [20]. Rapid relapses and poor outcome characterized the treatment of mantle cell lymphoma in the past [14]. The addition of ibrutinib has improved the outcome of treatment of mantle cell lymphoma in elderly patients with mantle cell lymphoma who may not tolerate full doses of antineoplastic agents [20]. Concerns about the risks of the treatment of mantle cell lymphoma led to the decision of the oncologist following our patients to postpone discussion of chemotherapy with the patient until the clinical manifestations of the disease, which were indolent early, worsened.    

## Conclusions

In PD patients, mantle cell lymphoma can cause cloudy peritoneal effluent with increased malignant cell count leading to difficulties in the distinction between noninfectious and infectious cloudy dialysate. These difficulties are recurrent and require special attention. The question whether malignancies causing the appearance of atypical cells in the peritoneal effluent affect the peritoneal transport mechanisms and consequently the performance of PD has not been addressed adequately and requires further study.
